# Life’s Essential 8 in Relation to Cardiovascular Disease and Mortality in Individuals With Diabetes

**DOI:** 10.1016/j.jacasi.2024.03.007

**Published:** 2024-05-21

**Authors:** Wenjuan Li, Aijun Xing, Wenqi Xu, Lu Guo, Xiang Gao, Shenghua Zhou, Jean-Philippe Drouin-Chartier, Shouling Wu, Zhangling Chen

**Affiliations:** a School of Clinical Medicine, North China University of Science and Technology, Tangshan, Hebei, China; b Department of Cardiology, Kailuan General Hospital, Tangshan, Hebei, China; c Department of Nutrition and Food Hygiene, School of Public Health, Institute of Nutrition, Fudan University, Shanghai, China; d Department of Cardiovascular Medicine, The Second Xiangya Hospital, Central South University, Changsha, Hunan, China; e Nutrition, Health and Society (NUTRISS) Research Center, Institute of Nutrition and Functional Foods (INAF), Laval University, Quebec City, Quebec, Canada; f Faculty of pharmacy, Laval University, Quebec City, Quebec, Canada; g Key Laboratory of Cardiometabolic Medicine in Hunan Province, Changsha, Hunan, China; h FuRong Laboratory, Changsha, Hunan, China; i Department of Epidemiology, Erasmus Medical Center, University Medical Center Rotterdam, Rotterdam, the Netherlands

**Keywords:** cardiovascular disease, cohort study, Life’s Essential 8, mortality

## Abstract

**Background:**

Evidence regarding the potential health effects of Life’s Essential 8 (LE8) score among individuals with type 2 diabetes (T2D) is limited.

**Objectives:**

The purpose of this study was to examine the associations of LE8 score with risk of cardiovascular disease (CVD) and mortality among individuals with T2D.

**Methods:**

We prospectively followed 19,915 Chinese participants with T2D at baseline or diagnosed during follow-up (Kailuan Study: 2006-2020), who were free of CVD at diagnosis of diabetes. Diet, lifestyle, and health conditions were repeatedly assessed every 2 years. The LE8 score (range 0-100), was calculated based on 8 components: diet quality, physical activity, smoking status, sleep health, body mass index, blood lipids, blood glucose, and blood pressure. We used time-varying cox models to model the associations.

**Results:**

During a median follow-up of 11.5 years in participants with T2D, there were 3,295 incident CVD cases and 3,123 deaths. Higher LE8 score was associated with lower risk of CVD incidence and total mortality among participants with diabetes. The multivariate-adjusted HRs for the highest quintile of LE8 score compared with the lowest quintile were 0.56 (95% CI: 0.53-0.59) for CVD, 0.57 (95% CI: 0.53-0.62) for heart disease, 0.53 (95% CI: 0.49-0.57) for stroke, and 0.73 (95% CI: 0.69-0.78) for total mortality (all P trend <0.001). Furthermore, compared with participants with stable or decreased LE8 score after diabetes diagnosis, those with increased LE8 score had 17% to 42% lower risk of CVD, heart disease, stroke, and mortality.

**Conclusions:**

A higher LE8 score was associated with a substantially lower risk of CVD incidence and total mortality among adults with T2D.

Type 2 diabetes (T2D) has become a global public health concern, with approximately 537 million adults living with diabetes worldwide in 2021.^1^ Cardiovascular disease (CVD) is the primary complication and is the leading cause of deaths in individuals with diabetes.^2^^,^^3^ It is thus important to identify and develop cost-effective strategies to prevent or delay the development of cardiovascular complications among individuals with T2D.

To further improve cardiovascular health (CVH) in general populations and to provide metrics for measuring and monitoring it, the American Heart Association (AHA) recently released an updated algorithm for evaluating CVH, called Life’s Essential 8 (LE8).^4^ Compared with the earlier developed Life’s Simple 7 (LS7), the LE8 is upgraded and enhanced by including an additional new component—sleep. As a result, the updated LE8 includes the 8 most important predictors of CVH: diet quality, physical activity, body mass index (BMI), smoking status, sleep health, blood pressure, blood glucose, and blood lipids. Accordingly, the scoring system of the LE8 is updated, with a score for each LE8 component ranging from 0 to 100 points, whereas each component in the LS7 score system ranged from 0 to 2 points.^4^^,^^5^ Earlier studies have shown that persons with an optimal level of LS7 score have lower risk of CVD and mortality,^6^, ^7^, ^8^, ^9^, ^10^ but recently, a few epidemiological studies have observed consistent beneficial associations of LE8 score and CVD risk among generally healthy populations.^11^, ^12^, ^13^, ^14^, ^15^ However, to our knowledge, very few studies have investigated the impact of LE8 score on risk of subsequent CVD events and mortality among high-risk individuals, such as those with diabetes, who have an elevated risk of developing CVD. Only 1 prospective study observed an inverse association of LE8 score with mortality among individuals with T2D, but it was mainly limited to baseline LE8 score use only.^16^ To date, there is no existing data specific for LE8 score in relation to CVD events among individuals with T2D. Furthermore, very little is known regarding whether improvement in LE8 score after diabetes diagnosis may yield benefits of CVH and overall health.

To fill these critical knowledge gaps, we prospectively investigated the associations of LE8 score with risk of subsequent CVD incidence, including heart disease and stroke, and total mortality among participants with T2D in a large prospective Chinese cohort with diet, lifestyle, and health data repeatedly measured every 2 years during a 14-year follow-up.

## Methods

### Study design and population

The Kailuan Study is an ongoing prospective cohort study of participants older than age 18 years from Kailuan Group in Tangshan, China. The study design and procedures were detailed previously.^17^ Briefly, 101,510 participants older than age 18 years from the Kailuan Group received health checkups and questionnaires in the Kailuan General Hospital and 10 affiliated hospitals and clinics in 2006. This study was further extended by including new individuals in 2008 (n = 24,540), 2010 (n = 9,118), 2012 (n = 17,981), 2014 (n = 9,088), 2016 (n = 4,112), and 2018 (n = 4,737). By the end of 2018, the Kailuan Study included a total of 171,086 participants. Upon entering the study, participants underwent a series of examinations in the hospitals and clinics every 2 years. All employees and retirees in the Kailuan Group were obliged to enroll in the Urban Employee Basic Medical Insurance, and drug treatment could be partly reimbursed by health insurance.

For the present study, we included 33,091 participants with prevalent T2D at baseline, as well as incident T2D cases diagnosed during follow-up through 2020. Participants were excluded if they had CVD at baseline, reported CVD before diabetes diagnosis during follow-up (n = 3,986), or had missing information on LE8 score (n = 9,190). After these exclusions, 19,915 participants with T2D remained in the final analyses, of whom 12,183 had prevalent T2D at baseline, and 7,732 had incident T2D develop during follow-up (Supplemental Figure 1). All participants provided written informed consent, and the Kailuan study was approved by the Ethics Committee of the Kailuan Medical Group.

### Assessment of LE8 score

We created LE8 score per the AHA’s LE8 algorithm.^4^ The LE8 score included 8 components: diet health, physical activity, smoking, sleep health, BMI, non–high-density lipoprotein (HDL) cholesterol, blood glucose, and blood pressure (Supplemental Table 1). The information on diet, physical activity, smoking, and sleep was collected using standardized self-reported questionnaires every 2 years since 2006. We used salt intake, tea consumption, and fatty foods to measure diet quality, which have been previously reported to be associated with CVD risk in Chinese people, as done previously.^18^^,^^19^ Weight, height, blood lipids, blood glucose, and blood pressure were measured at the medical examination center of the Kailuan Group every 2 years since 2006. BMI was calculated by dividing weight (kg) by the square of height (m). Blood samples were collected in the morning following an 8- to 12-h overnight fast at each visit. The fasting blood glucose, total cholesterol, low-density lipoprotein cholesterol, and HDL concentrations were measured using a Hitachi 7600 autoanalyzer (Tokyo, Japan) at the central laboratory of Kailuan General Hospital. The information and scoring algorithm for each component is provided in Supplemental Table 1. The range of scoring algorithm for each component was 0 to 100. The overall LE8 score was calculated by summing the scores for the 8 components and dividing them by 8, which thus ranged from 0 (worst health) to 100 (best health).^4^ We categorized the overall LE8 score into 5 quintiles for the main analyses.^4^

### Assessment of outcomes

The primary outcomes of our study included CVD incidence and total mortality. Incident CVD was defined as heart disease (including myocardial infarction, atrial fibrillation, and heart failure) and stroke. The information of CVD diagnosis was obtained from the Municipal Social Insurance Institution and Hospital Discharge Register and was updated annually during the follow-up period, which covered all the Kailuan study participants. An expert panel collected and reviewed the annual discharge records from 11 local hospitals to identify individuals who were suspected of having CVD. The diagnosis of myocardial infarction was determined by the patient’s clinical symptoms, electrocardiography findings, and dynamic changes in myocardial enzymes according to the World Health Organization’s Multinational Monitoring of Trends and Determinants in CVD criteria.^20^ Atrial fibrillation diagnoses were retrieved from discharge registers at the municipal social insurance that covered all participants from the Kailuan Study and resting electrocardiogram of each survey. The electrocardiogram and diagnosis were completed by 2 professional electrocardiologists according to the European Society of Cardiology guidelines.^21^ Incident heart failure was defined according to the criteria of the European Society of Cardiology on the basis of clinical symptoms, echocardiography, chest x-ray, and electrocardiography.^22^ Stroke was diagnosed based on neurological signs, clinical symptoms, and neuroimaging tests, including computed tomography or magnetic resonance imaging per the World Health Organization criteria.^23^ Total mortality data were collected from provincial vital statistics offices and reviewed by physicians.

### Covariates

Information on anthropometric, lifestyle, and socioeconomic factors was assessed through biennial questionnaires every 2 years since 2006, including age, smoking, alcohol consumption, education level, income, marital status, and family history of CVD and diabetes.

### Statistical analyses

Person-time was calculated from the date of a diabetes diagnosis to occurrence of study outcomes, last return of a valid follow-up questionnaire, or the end of follow-up (December 2020), whichever came first. Time-varying cox proportional hazards models were used to estimate HRs and 95% CIs for the associations of LE8 score with risk of subsequent total CVD, heart disease, and stroke incidence, and total mortality among individuals with diabetes. We modeled LE8 score as a time-varying variable. We also included age, sex, alcohol drinker, income, education, marital status, family history of diabetes, and family history of CVD as covariates in the analyses, of which, alcohol drinker, income, marital status, family history of diabetes, and family history of CVD were considered as time-varying covariates. All of the covariates were selected based on whether they may be highly correlated with the exposures and outcomes and may bias the association according to previous studies. We further tested the statistical significance of linear trends by modelling the median value within each quintile of LE8 score as a continuous variable and then examining the significance of this variable. We examined the individual score for each component of LE8 with these outcomes. The R codes followed the steps suggested by Therneau et al^24^ and Zhang et al.^25^

In a secondary analysis, we explored the associations of changes in LE8 score after diabetes diagnosis with CVD incidence and total mortality. Changes in LE8 score after diabetes diagnosis were defined as the absolute difference in LE8 score from baseline for prevalent T2D or the date of diabetes diagnosis for incident T2D to occurrence of outcomes or the end of follow-up. In this analysis, we adjusted for age, sex, education, initial and changes in alcohol drink status, initial and changes in income, initial and changes in marital status, family history of diabetes, family history of CVD, and initial LE8 score.

Furthermore, we stratified analyses by age, sex, BMI, follow-up time, smoking status, alcohol consumption, and family history of diabetes or CVD. We used a Bonferroni-corrected *P* value threshold (0.05/7 = 0.007) to account for multiple comparisons in the interaction tests.

### Sensitivity analysis

We conducted several sensitivity analyses to test the robustness of our findings based on fully adjusted models (model 2). First, we excluded blood glucose from LE8 score because individuals with diabetes were included. Second, analyses were restricted to participants with T2D diagnosed during follow-up, where we additionally adjusted for diabetes duration given that CVD risk profiles may differ between prevalent diabetes cases and incident cases, and only incident cases had data on diabetes duration (n = 7,732). Third, we additionally adjusted for hyperlipidemia, hypertension, and hyperglycemia as well as lipid-lowering medications, antihypertensive medications, and glucose-lowering medications. Fourth, we excluded cancer cases at baseline and follow-up and repeated the analyses (n = 18,827), because participants with cancers may substantially change diet and lifestyle over time. Fifth, we excluded CVD cases and deaths within the first 4 years of follow-up and analyses were repeated (n = 18,452). Sixth, according to the AHA’s recommendations, we divided LE8 score into 3 categories (low: <50; moderate: 50 to <80; high: ≥80)^4^ as well as created the LS7 score and divided it into 3 categories (poor: 0-7; intermediate: 8-11; ideal: 12-14)^5^ to compare the effect sizes of the LE8 and LS7 among individuals with T2D. Finally, we calculated C-index to assess the ability of the model of LE8 score with adjusted covariates for predicting incident CVD event risk and total mortality.

All statistical analyses were performed with SAS software version 9.4 (SAS Institute Inc) and R version 4.2.1 (R Foundation for Statistical Computing). A *P <* 0.05 (2-sided) was considered statistically significant unless otherwise specified.

## Results

### Population characteristics

Table 1 shows baseline characteristics of the participants by quintiles of LE8 score. At baseline, participants with higher LE8 score were more likely to be women, to not be alcohol drinkers, and to have a higher level of educational attainment. Supplemental Table 2 presents individual components of LE8 across the quintiles of LE8 score.

**Table 1:** Baseline Characteristics by Quintiles of Baseline LE8 Score

	Total (N = 19,915)	Q1 (n = 4,046)	Q2 (n = 3,774)	Q3 (n = 4,301)	Q4 (n = 3,807)	Q5 (n = 3,987)
LE8 score points	56.4 ± 10.1	42.0 ± 5.1	51.2 ± 1.6	56.7 ± 1.6	62.0 ± 1.6	70.2 ± 4.3
Participant age, y	55.3 ± 10.7	53.7 ± 10.0	54.8 ± 10.4	55.6 ± 10.4	56.0 ± 10.8	56.2 ± 11.6
Male, %	84.1	91.3	86.0	84.3	80.9	77.7
Alcohol-drinker, %						
Never	61.3	39.4	54.9	65.9	70.7	75.7
Past	3.3	4.1	3.7	3.3	2.9	2.6
Current	35.4	56.5	41.4	30.9	26.3	21.7
Education, %						
Illiteracy/elementary	12.1	14.2	13.1	10.2	11.3	11.7
Middle school	82.6	81.1	82.0	84.6	83.3	81.9
College/university	5.3	4.7	4.9	5.3	5.4	6.3
Score points for each component of LE8						
Diet quality score	40.9 ± 16.7	36.8 ± 16.4	39.9 ± 16.3	40.3 ± 15.2	42.3 ± 16.2	45.2 ± 18.3
Physical activity score	51.6 ± 30.6	33.6 ± 30.8	47.5 ± 30.2	53.0 ± 27.1	58.0 ± 27.5	66.3 ± 27.1
Smoking score	70.7 ± 35.6	45.3 ± 31.9	61.9 ± 36.2	74.6 ± 34.7	81.6 ± 31.3	90.3 ± 24.3
Sleep health score	86.6 ± 22.7	74.2 ± 28.5	84.9 ± 23.2	89.5 ± 20.1	90.8 ± 18.8	93.5 ± 15.4
BMI score	60.3 ± 23.7	48.9 ± 21.1	54.4 ± 21.8	58.1 ± 21.8	64.9 ± 22.0	75.3 ± 22.6
Blood lipids score	62.8 ± 31.1	43.3 ± 28.6	54.2 ± 29.1	63.7 ± 29.6	68.9 ± 27.7	83.7 ± 24.4
Blood glucose score	35.2 ± 18.0	30.9 ± 15.9	33.3 ± 16.7	34.8 ± 16.3	36.1 ± 18.3	40.8 ± 21.0
Blood pressure score	43.2 ± 33.3	23.1 ± 28.1	33.2 ± 31.1	39.7 ± 31.8	53.6 ± 29.6	66.8 ± 26.6

Values are mean ± SD for continuous variables, or percentages for categorical variables. An overall Life’s Essential 8 (LE8) score ranges from 0-100. Scores for each component of LE8 also range from 0-100.

BMI = body mass index.

### Main results

During a median of 11.5 years of follow-up in participants with T2D, we documented 3,295 incident CVD cases and 3,123 deaths. After multivariable adjustments, including age, sex, education, income, marital status, alcohol intake, and family history of diabetes and CVD, higher LE8 score was associated with lower risk of subsequent CVD incidence and total mortality among participants with T2D (Table 2). Compared with those in the lowest quintile of LE8 score, the participants with T2D in the highest quintile had a 44% (41%, 47%) lower risk of CVD, a 43% (38%, 47%) lower risk of heart disease, a 47% (43%, 51%) lower risk of stroke, and a 27% (22%, 31%) lower risk of total mortality (all *P* trend <0.001). Each 10-point increment of LE8 score was associated with a 17% (15%, 18%), 15% (13%, 17%), 19% (17%, 20%), and 10% (9%, 12%) lower risk for CVD, heart disease, and stroke incidence, as well as total mortality, respectively (Central Illustration). Further, a higher score of each individual component, which reflected higher diet quality, more physical activity, higher sleep quality, less smoking, lower BMI, lower blood glucose, lower non-HDL cholesterol, or lower blood pressure, was associated with lower risk of total CVD, heart disease, stroke incidence, or total mortality (Supplemental Table 3).

**Table 2:** Associations of Time-Varying LE8 Score With Risk of CVD and Mortality Among Individuals With T2D

	Q1	Q2	Q3	Q4	Q5	*P* Value for Trend	Per 10-Point Increase
LE8 score points (median)	43.5	52.1	57.9	63.8	72.0		
CVD							
No. of cases/person-years	837/39,473	687/36,953	747/42,779	571/37,970	453/40,419		
Model 1	1.00 (Ref)	0.87 (0.83-0.92)	0.80 (0.76-0.84)	0.72 (0.68-0.75)	0.57 (0.54-0.60)	<0.001	0.84 (0.83-0.85)
Model 2	1.00 (Ref)	0.87 (0.83-0.91)	0.79 (0.75-0.83)	0.70 (0.67-0.74)	0.56 (0.53-0.59)	<0.001	0.83 (0.82-0.85)
Heart disease							
No. of cases/person-years	436/41,389	371/38,452	396/44,329	313/39,044	235/41,425		
Model 1	1.00 (Ref)	0.88 (0.82-0.94)	0.82 (0.77-0.88)	0.77 (0.72-0.83)	0.60 (0.56-0.65)	<0.001	0.86 (0.84-0.88)
Model 2	1.00 (Ref)	0.86 (0.81-0.92)	0.80 (0.75-0.86)	0.74 (0.69-0.79)	0.57 (0.53-0.62)	<0.001	0.85 (0.83-0.87)
Stroke							
No. of cases/person-years	465/41,215	368/38,326	406/44,354	297/39,129	240/41,293		
Model 1	1.00 (Ref)	0.85 (0.80-0.91)	0.76 (0.71-0.81)	0.65 (0.61-0.70)	0.52 (0.49-0.56)	<0.001	0.81 (0.80-0.83)
Model 2	1.00 (Ref)	0.85 (0.80-0.91)	0.76 (0.71-0.81)	0.66 (0.61-0.70)	0.53 (0.49-0.57)	<0.001	0.81 (0.80-0.83)
Total morality							
No. of case/person-years	611/43,326	638/39,976	678/46,045	581/40,329	615/42,350		
Model1	1.00 (Ref)	0.89 (0.84-0.94)	0.83 (0.79-0.88)	0.78 (0.74-0.83)	0.72 (0.68-0.76)	<0.001	0.89 (0.88-0.91)
Model 2	1.00 (Ref)	0.90 (0.85-0.95)	0.84 (0.80-0.89)	0.79 (0.75-0.84)	0.73 (0.69-0.78)	<0.001	0.90 (0.88-0.91)

Values are HR (95% CI) unless otherwise indicated. Model 1: age (years), and sex (male, female). Model 2: Model 1 + education (illiteracy or elementary, middle school, college/university), income (< median, ≥ median), marital status (yes, no), alcohol drinker (never, past, current), family history of diabetes (yes, no), and family history of cardiovascular disease (CVD) (yes, no).

LE8 = Life’s Essential 8; T2D = type 2 diabetes.

**Central Illustration:**
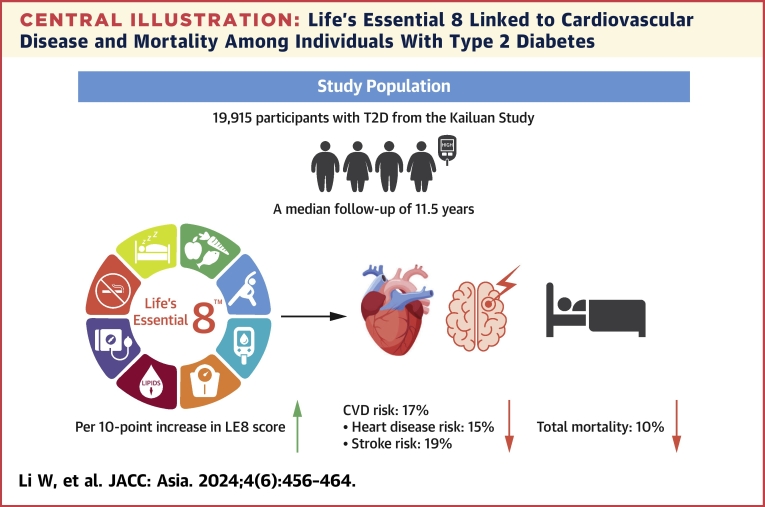
Life’s Essential 8 Linked to Cardiovascular Disease and Mortality Among Individuals With Type 2 Diabetes Among 19,195 participants with T2D, each 10-point increase in LE8 score was associated with a 17% lower risk of CVD, a 15% lower risk of heart disease, a 19% lower risk of stroke, and a 10% lower risk of total mortality. Multivariable time-varying cox models were adjusted for age (years), sex (male, female), education (illiteracy or elementary, middle school, college/university), income (< median, ≥ median), marital status (yes, no), alcohol-drinker (never, past, current), family history of diabetes (yes, no), and family history of CVD (yes, no). CVD was defined as heart disease and stroke. CVD = cardiovascular disease; LE8 = Life’s Essential 8; T2D = type 2 diabetes.

**Figure 1:**
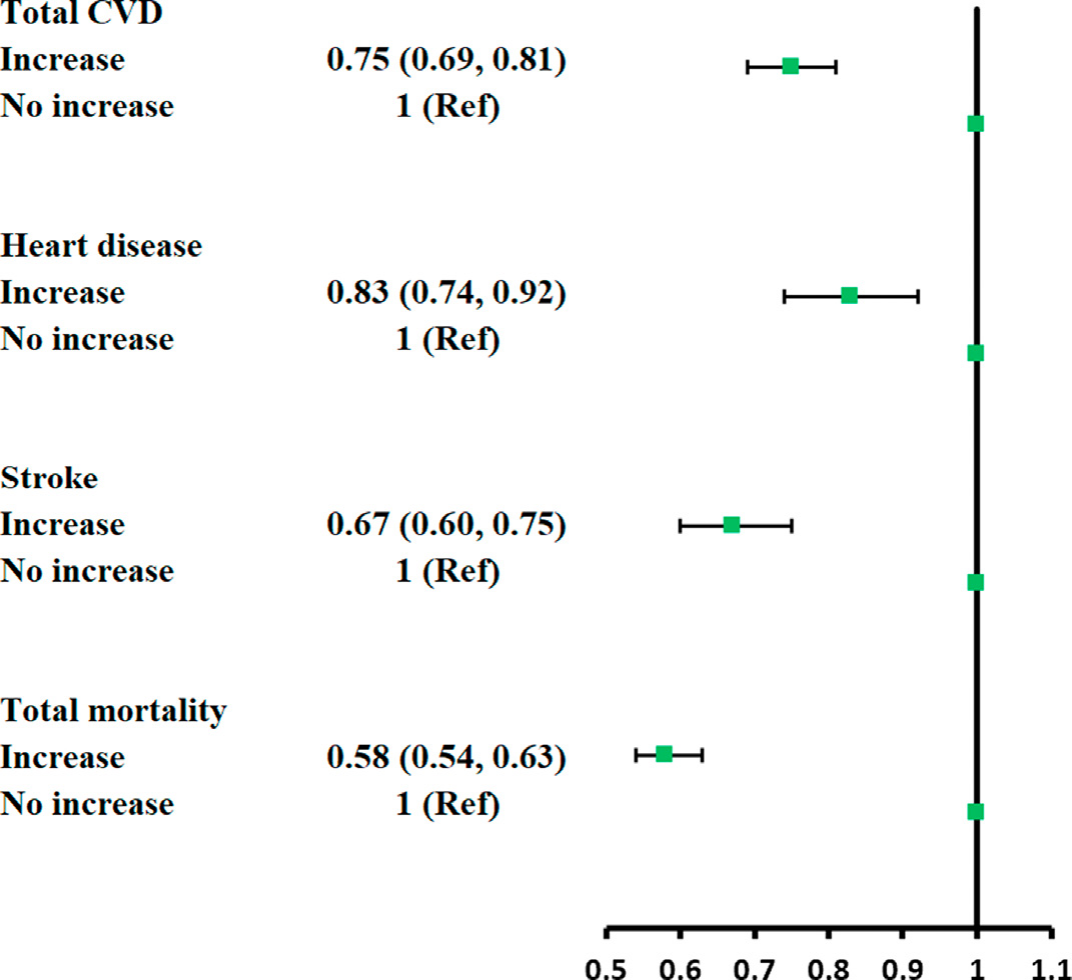
Changes in Life’s Essential 8 With CVD and Mortality After Diabetes Diagnosis Multivariable analyses were adjusted for age (years), sex (male, female), education (illiteracy or elementary, middle school, college/university), initial income (< median, ≥ median) and changes in income (< median always, ≥ median always, change from < median to ≥ median, change from ≥ median to < median), initial marital status (yes, no), and changes in marital status (always single, always married, change from married to single, change from single to married), initial alcohol drinker (yes, no), changes in alcohol-drinker (always drinker, always nondrinker, change from drinker to nondrinker, change from nondrinker to drinker), family history of diabetes or cardiovascular disease (CVD) (yes, no), and initial LE8 score (continuous). No increase group included participants with stable or decreased LE8 score after diabetes diagnosis.

In a secondary analysis, increment in LE8 score after diabetes diagnosis over time was significantly associated with a lower risk of subsequent CVD, heart disease, and stroke incidence, as well as total mortality (Figure 1). Compared with participants with stable or decreased LE8 score after diabetes diagnosis, those who increased the score had a 25% (19%, 31%) lower risk for CVD, a 17% (8%, 26%) lower risk for heart disease, a 33% (25%, 40%) lower risk for stroke, and a 42% (37%, 46%) lower risk for total mortality. These inverse associations persisted in subgroup analysis stratified by age, sex, BMI, follow-up time, smoking status, alcohol consumption, and family history of diabetes or CVD. None of the interaction tests was statistically significant, except for the interaction of LE8 score and age for CVD risk (*P* interaction <0.001) (Supplemental Table 4).

### Sensitivity analysis results

In sensitivity analyses, the results remained similar when we excluded blood glucose from LE8 score (Supplemental Table 5); when we additionally adjusted for hypertension, hyperlipidemia, and hyperglycemia as well as antihypertensive medications, lipid-lowering medications, and glucose-lowering medications, or additionally adjusted for diabetes duration (Supplemental Tables 6 and 7); and when we excluded cancer cases at baseline and follow-up or excluded CVD cases and deaths that occurred within the first 4 years of follow-up (Supplemental Tables 8 and 9). Further, the effect sizes of the associations of LE8 score with the study outcomes were generally stronger than those of LE7 score (Supplemental Table 10). The participants with T2D in the high category of LE8 score (≥80 points) had a 34% to 68% lower risk for CVD, heart disease, stroke, and total mortality, whereas those in the ideal group of LS7 score had a 33% to 38% lower risk for these outcomes. Finally, the model of LE8 score with adjusted covariates yielded reasonable discrimination. The C-index was 0.63 (0.62, 0.64) for CVD risk and 0.74 (0.73, 0.75) for total mortality.

## Discussion

In this prospective cohort among Chinese people with diabetes, higher LE8 score was associated with lower risk of CVD, heart disease, and stroke incidence, as well as total mortality. These associations were independent of several risk factors, including age, sex, education, income, marital status, alcohol intake, and family history of diabetes and CVD. In addition, greater increase in LE8 score after diabetes diagnosis over time was significantly associated with a lower risk of CVD and total mortality.

### Comparison with previous studies

Only a few recent prospective cohort studies including our previous analyses have examined associations of LE8 score with CVD risk and mortality among generally healthy populations, and observed consistently beneficial associations.^11^^,^^12^^,^^15^ However, data regarding the potential impact of LE8 score on health outcomes among persons with diabetes are sparse. Only a recent study has investigated the association of LE8 score and mortality among diabetes and reported an inverse association, but with a main limitation of baseline LE8 score use only.^16^ Yet, to date, no studies have examined associations of LE8 score with risk of CVD events among individuals with diabetes. Leveraging the large sample size and repeated measurements of LE8 score and covariates every 2 years, we addressed the main limitation and confirmed the inverse association with total mortality. Further, we made some novel observations. We observed that higher LE8 score was associated with a lower risk of CVD events among these participants with T2D. This is the first study to examine the associations of LE8 score with risk of subsequent CVD events among diabetes. Of note, the effect sizes of these associations among individuals with T2D were somewhat stronger for CVD risk and a bit weaker for total mortality than those in previous analyses among healthy populations. For example, in our analysis of individuals with T2D, we observed a 17% lower risk of each 10-point increase of LE8 score for CVD risk and a 27% lower risk for total mortality in the highest quintile of LE8 score. Petermann-Rocha et al^14^ observed a 9.2% lower risk of each 10 points increase for CVD, and Isiozor et al^26^ observed a 47% lower risk for total mortality in the highest quartile of LE8 score among generally heathy populations. These findings have indicated that adhering to LE8 score may be beneficial for promoting CVD health and overall health among not only generally healthy people but also people with diabetes.

Further, in the current analysis, we observed that increased LE8 score after diabetes diagnosis was associated with lower risk of subsequent CVD incidence and mortality after controlling for initial LE8 score, indicating that adherence to LE8 score may prevent development of CVD risk and death, irrespective of initial CVH among individuals with T2D. Also, to explore whether adherence to LE8 score may be more beneficial for promoting CVH among individuals with T2D than adherence to LS7 score, we examined the associations of LS7 score with the study outcomes in the same participants and compared the effect sizes of LE8 score and LS7 score. We found that the effect sizes of the associations of LE8 score were generally stronger than those of LS7 score, indicating that the adherence to LE8 score may be more beneficial for improving CVH among individuals with T2D than adherence to LS7 score. In addition, we examined the associations of each individual score of LE8 components with the outcomes among these patients, and our results were in line with previous cohorts where multi-components of LE8, such as higher diet quality, and higher sleep quality were associated with the lower risk among individuals with diabetes.^27^, ^28^, ^29^ In particular, the inverse association of sleep health with the outcomes in our analyses further supported the importance of adding sleep quality to LE8 for improving CVH. A recent meta-analysis of randomized controlled trials, including 16,574 patients with prediabetes and T2D, observed no beneficial effects of lifestyle interventions on CVD health.^30^ The reasons for the discrepancies between the meta-analysis of randomized controlled trials and the cohort studies remained unclear, but we noticed that randomized controlled trials generally had a relatively short intervention duration (mean 4.25 years).

### Study strengths and limitations

The strengths of the present study include its prospective design; relatively large sample size; and long-term follow-up with repeated assessments of dietary, lifestyle, and health condition variables every 2 years.

Several limitations should be considered. First, measurement errors were inevitable in the estimates of some self-reported dietary and lifestyle variables, such as food intakes. However, such measurement errors were likely to be nondifferential in this prospective study and thus would be more likely to bias the associations toward the null. Second, because of limited data on more specific dietary factors in our cohort, we could not calculate an overall diet quality score, such as alternative healthy eating index to assess diet quality. Yet, we mainly focused on LE8 score rather than diet quality. Therefore, we used dietary salt, fatty foods, and tea consumption to reflect diet quality, which are the most primary dietary factors in relation to CVH among Chinese people.^31^ Third, our study participants were mainly comprised of staff of Kailuan Group of Chinese ancestry, which limits the generalizability of our findings to other racial/ethnic or socioeconomic groups. Fourth, we were unable to account for other cause-specific deaths as competing risk events because of the unavailability of data. Fifth, we excluded the participants with diabetes but without LE8 score, which might have resulted in selection bias. Finally, it was difficult to rule out residual and unmeasured confounding, despite the adjustment for important personal and lifestyle factors.

## Conclusions

Among Chinese people with T2D, higher LE8 score was associated with lower CVD incidence and mortality. Further, increased LE8 score after diabetes diagnosis over time was associated with lower risk of CVD incidence and mortality. Taken together, our findings suggest that greater adherence to LE8 score may facilitate the prevention of CVD complications and premature deaths among adults with T2D.

Perspectives **COMPETENCY IN MEDICAL KNOWLEDGE:** The present study showed that higher LE8 score was associated with lower risk of CVD, heart disease, and stroke incidence and lower mortality among individuals with diabetes.

**TRANSLATIONAL OUTLOOK:** These findings highlighted that the LE8 score has the potential to serve as a personal guide for CVH and overall health promotion strategies in individuals with T2D. More research needs to be done in other races and ethnicities.

## Funding Support and Author Disclosures

This work was supported by the Kailuan General Hospital internal grant, as well as the National Natural Science Foundation of China (No. 82304148 to Dr Chen) and grant from The Scientific Research Program of FuRong Laboratory to (No. 2023SK2017-1 to Dr Chen). The funders had no role in study design, data collection and analysis, decision to publish, or preparation of the manuscript. All other authors have reported that they have no relationships relevant to the contents of this paper to disclose.
