# Supplementary material for: Life’s Essential 8 in Relation to Cardiovascular Disease and Mortality in Individuals With Diabetes
Source: JACC Asia. 2024 May 21;4(6):456–64. doi: 10.1016/j.jacasi.2024.03.007 (PMC11291351; doi:10.1016/j.jacasi.2024.03.007)

**Supplemental Table 1. Definition of Life’s Essential 8 by AHA and the criteria used in this study**

| Components | Metric | Method of measurement | Quantification of CVH metric |
| --- | --- | --- | --- |
| Health behaviors | Diet health | Measurement: Self-reported intake of salt, fatty foods, and tea collected using food frequency questionnaires  Examples of salt intake measurement: “What flavor do you prefer.”  Examples of fatty food intake measurement: “How often do you eat fatty foods, such as red and processed foods, deep-fried foods?”  Example of tea intake measurement: “How often do you drink tea?” | Metric: The unweighted average of salt, fatty food, and tea scoring.  Salt scoring:  Points Level  100 <6 g/day  50 6-12 g/day  0 >12 g/day  Fatty food scoring:  Points Level  100 <1 time/week  50 1-3 times/week  0 >3 times/week  Tea scoring:  Points Level  100 ≥4 times/week  75 1-3 times/week  50 1-3 times/month  25 < 1 time/month  0 Never |
|  | Physical activity | Measurement: Self-reported times of physical activity per week.  Example tools for measurement: “How many times did you usually spend on physical activity (note: It took at least 20 minutes each time)? ” | Metric: Minutes of physical activity per week.  Scoring:  Points Level  100 ≥ 60  50 20-60  0 <20 |
|  | Nicotine exposure | Measurement: Self-reported use of cigarettes  Example tools for measurement: Do you now smoke cigarettes? (Never smoker, former smoker, some days, every day) | Metric: Smoking status  Scoring:  Points Status  100 Never smoker  50 Former smokers quit ≥ 1y  25 Current smokers, < 1cigarette/d  0 Current smoker, ≥1cigarette/d |
|  | Sleep health | Measurement: Self-reported average hours of sleep per night  Example tools for measurement: “On average, how many hours of sleep do you get per night?” | Metric: Average hours of sleep per night  Scoring:  Points Level  100 7 - <9 h  90 9 - < 10 h  70 6 - < 7 h  40 5 - <6 or ≥10 h  20 4 - <5 h  0 <4h |
| Health factors | Body mass index | Measurement: Body weight (kg) divided by height squared(m²)  Example tools for measurement: Objective measurement of height and weight | Metric: Body mass index (kg/m2)  Scoring:  Points Level  100 <23  75 23.0-24.9  50 25.0-29.9  25 30.0-34.9  0 ≥35.0 |
|  | Blood lipids | Measurement: Plasma total and HDL cholesterol with the calculation of non-HDL cholesterol.  Example tools for measurement: Fasting blood sample.  non-HDL-cholesterol unit conversion:  1mmol/L= 38.67mg/L  1mg/L=0.02586mmol/L | Metric: Non-HDL cholesterol (mmol/L)  Scoring:  Points Level  100 < 3.36  60 3.36-4.13  40 4.14-4.90  20 4.91-5.68  0 ≥ 5.69  If drug treated, subtract 20 points |
|  | Blood glucose | Measurement: Fasting blood glucose(FBG)  Example tools for measurement: Fasting blood glucose sample.  HBA1C to FBG (mg/L) to conversion:  28.7 * A1C - 46.7 = FBG  FBG unit conversion:  1mg = 0.056mmol/L  1mmol/L= 18.02 mg/dL | Metric: FBG (mmol/L)  Scoring:  Points Level  100 No history of diabetes with FBG <5.6  60 No diabetes with FBG 5.6-6.9  40 Diabetes with FBG < 8.6  30 Diabetes with FBG 8.6-10.1  20 Diabetes with FBG 10.2-11.6  10 Diabetes with FBG 11.7-13.2  0 Diabetes with FBG ≥13.3 |
|  | Blood pressure | Measurement: Appropriately measured systolic and diastolic blood pressure  Example tools for measurement: Corrected Mercury sphygmomanometer | Metric: Systolic and diastolic blood pressure (mm Hg)  Scoring:  Points Level  100 <120 / < 80  75 120-129 / < 80  50 130-139 or 80-89  25 140-159 or 90-99  0 ≥160 or ≥100  If drug treated, subtract 20 points. |

Data on the 8 components were repeatedly assessed every 2 years. CVH, cardiovascular health.

**Supplemental Table 2. Individual components of LE8 across quintiles of time-varying LE8 score**

| **Characteristics** | **Total** | **Q1** | **Q2** | **Q3** | **Q4** | **Q5** |
| --- | --- | --- | --- | --- | --- | --- |
|  | (n=19,915) | (n=4,046) | (n=3,774) | (n=4,301) | (n=3,807) | (n=3,987) |
| **LE8 score points** | 56.4 (10.1) | 42.0 (5.1) | 51.2 (1.6) | 56.7 (1.6) | 62.0 (1.6) | 70.2 (4.3) |
| **Tea consumption (%)** |  |  |  |  |  |  |
| -Never or less than once/month | 79.4 | 79.0 | 79.1 | 81.5 | 80.3 | 77.0 |
| -1 to 3 times/month | 5.3 | 5.9 | 6.1 | 4.8 | 4.9 | 4.9 |
| -1 to 3 times/week | 5.0 | 4.5 | 4.5 | 4.9 | 5.0 | 6.2 |
| -≥4 times/week | 10.3 | 10.6 | 10.3 | 8.8 | 9.8 | 11.9 |
| **Fatty food consumption^*^ (%)** |  |  |  |  |  |  |
| -＜1 times/week | 14.3 | 12.6 | 14.2 | 12.6 | 14.7 | 17.8 |
| -1-3 times/week | 77.2 | 71.4 | 75.8 | 80.7 | 79.7 | 78.0 |
| -＞3 times/week | 8.5 | 16.1 | 10.0 | 6.6 | 5.5 | 4.2 |
| **Salt intake (%)** |  |  |  |  |  |  |
| -＜6 g/day | 13.2 | 9.0 | 12.0 | 11.4 | 14.7 | 18.9 |
| **Physical activity level^†^ (%)** |  |  |  |  |  |  |
| - Never | 17.8 | 41.0 | 21.4 | 12.6 | 9.3 | 4.6 |
| -＜3 times/ week | 65.3 | 52.2 | 65.3 | 73.1 | 70.9 | 64.8 |
| -＞3 times/week | 16.9 | 6.9 | 13.3 | 14.3 | 19.8 | 30.6 |
| **Smoke status (%)** |  |  |  |  |  |  |
| -Never | 59.1 | 24.6 | 46.7 | 64.5 | 73.9 | 85.8 |
| -Past | 5.6 | 7.2 | 7.5 | 4.8 | 4.8 | 3.7 |
| -Current | 35.3 | 68.2 | 45.8 | 30.7 | 21.3 | 10.5 |
| **Sleep duration (%)** |  |  |  |  |  |  |
| -＜6 h/day | 10.2 | 22.0 | 11.8 | 7.3 | 6.2 | 3.7 |
| -6-9 h/day | 88.9 | 77.1 | 87.2 | 92.0 | 93.0 | 95.4 |
| -＞9 h | 0.9 | 0.9 | 1.0 | 0.7 | 0.8 | 1.0 |
| **BMI, kg/m²** | 26.2 (3.5) | 27.7 (3.5) | 27.0 (3.4) | 26.4 (3.3) | 25.6 (3.2) | 24.4 (3.2) |
| **Blood lipid, mmol/L** |  |  |  |  |  |  |
| -TC, mmol/L | 5.3 (1.5) | 6.0 (1.7) | 5.5 (1.9) | 5.2 (1.2) | 5.0 (1.0) | 4.6 (1.1) |
| -HDL, mmol/L | 1.5 (0.5) | 1.5 (0.5) | 1.5 (0.5) | 1.5 (0.5) | 1.5 (0.5) | 1.5 (0.6) |
| **Blood glucose, mmol/l** | 8.9 (4.4) | 9.4 (4.3) | 9.2 (4.9) | 8.8 (3.8) | 8.8 (4.6) | 8.2 (4.1) |
| **Blood pressure, mmHg** |  |  |  |  |  |  |
| -SBP | 138.7 (20.7) | 148.5 (20.2) | 143.6 (20.5) | 140.7 (20.1) | 133.4 (18.5) | 126.9 (16.8) |
| -DBP | 86.9 (11.7) | 93.8 (11.8) | 89.9 (11.1) | 87.6 (10.9) | 83.6 (9.7) | 79.6 (9.0) |

Values are means (SD) for continuous variables or percentages for categorical variables. LE8, Life’s Essential 8; SBP, systolic blood pressure; DBP, diastolic blood pressure; BMI, body mass index; TC, total cholesterol; HDL, high density lipoprotein cholesterol. ^*^Fatty foods in the Kailuan Study included red and processed meat, fried foods, fast foods, fatty snacks, animal-fat foods and so on. ^†^It took at least 20 minutes each time.

**Supplemental Table 3. Associations of individual components of time-varying LE8 with CVD and mortality among individuals with T2D^*^**

| **Each 20 points increase** | **CVD** | **Heart disease** | **Stroke** | **Total morality** |
| --- | --- | --- | --- | --- |
| **Diet quality score** | 0.97 (0.96, 0.99) | 0.97 (0.95, 0.99) | 0.97 (0.95, 0.99) | 0.95 (0.94, 0.97) |
| **Physical activity score** | 0.98 (0.97, 0.99) | 0.99 (0.98, 1.00) | 0.97 (0.96, 0.98) | 0.93 (0.92, 0.94) |
| **Smoking score** | 0.97 (0.96, 0.97) | 0.96 (0.95, 0.98) | 0.96 (0.95, 0.97) | 0.98 (0.97, 0.99) |
| **Sleep health score** | 0.97 (0.95, 0.98) | 0.96 (0.94, 0.98) | 0.98 (0.96, 0.997) | 0.91 (0.90, 0.92) |
| **BMI score** | 0.98 (0.97, 0.995) | 0.96 (0.95, 0.98) | 1.00 (0.98, 1.02) | 1.00 (0.99, 1.02) |
| **Blood lipids score** | 0.97 (0.96, 0.98) | 0.98 (0.97, 0.99) | 0.96 (0.95, 0.97) | 0.99 (0.98, 1.00) |
| **Blood glucose score** | 0.91 (0.90, 0.92) | 0.93 (0.91, 0.95) | 0.89 (0.87, 0.91) | 0.91 (0.89, 0.92) |
| **Blood pressure score** | 0.90 (0.89, 0.91) | 0.93 (0.92, 0.94) | 0.88 (0.87, 0.89) | 0.98 (0.97, 0.99) |
| Data were HRs and 95%CIs among individuals with T2D. Multivariable time-varying cox models were adjusted for age (years), sex (male, female), education (illiteracy or elementary, middle school, college/university), income (< median, ≥ median), marital status (yes, no), alcohol-drinker (never, past, current), family history of diabetes (yes, no), and family history of CVD (yes, no). ^*^Higher diet quality score, higher physical activity score, higher smoking score, higher sleep health score, higher BMI score, higher blood lipids score, higher blood glucose score, and higher blood pressure score reflected higher diet quality, more physical activity, less smoking, higher sleep health quality, lower BMI, lower blood non-HDL cholesterol, lower blood glucose, and lower blood pressure, respectively. CVD, cardiovascular disease; LE8, Life’s Essential 8; T2D, type 2 diabetes. | | | | |
|  |  |  |  |  |
|  |  |  |  |  |

**Supplemental Table 4. Stratified hazard ratios of CVD and mortality per time-varying LE8 score by characteristics of participants**

| **Life’ essential 8 score** | | | | | | | |
| --- | --- | --- | --- | --- | --- | --- | --- |
|  | **Q1** | **Q2** | **Q3** | **Q4** | **Q5** | **Per 10 points increase** | **P_interaction_** |
|  |  |  |  |  |  |  |  |
| **CVD** | | | | | | | |
| **Age** |  |  |  |  |  |  |  |
| <65 years | 1 (Ref) | 0.85 (0.81, 0.90) | 0.76 (0.72, 0.80) | 0.67 (0.63, 0.71) | 0.53 (0.50, 0.56) | 0.82 (0.81, 0.83) | <0.001 |
| ≥65 years | 1 (Ref) | 0.97 (0.86, 1.10) | 0.94 (0.83, 1.05) | 0.89 (0.79, 1.01) | 0.71 (0.63, 0.81) | 0.90 (0.87, 0.93) |  |
| **Sex** |  |  |  |  |  |  |  |
| Female | 1 (Ref) | 0.79 (0.70, 0.90) | 0.71 (0.62, 0.80) | 0.57 (0.50, 0.65) | 0.48 (0.42, 0.54) | 0.79 (0.76, 0.82) | 0.29 |
| Male | 1 (Ref) | 0.88 (0.83, 0.93) | 0.80 (0.76, 0.84) | 0.73 (0.69, 0.78) | 0.58 (0.55, 0.61) | 0.84 (0.83, 0.86) |  |
| **BMI** |  |  |  |  |  |  |  |
| <25kg/m^2^ | 1 (Ref) | 0.92 (0.83, 1.01) | 0.80 (0.73, 0.88) | 0.77 (0.70, 0.84) | 0.57 (0.52, 0.62) | 0.82 (0.80, 0.84) | 0.03 |
| ≥25kg/m^2^ | 1 (Ref) | 0.86 (0.81, 0.91) | 0.80 (0.75, 0.85) | 0.70 (0.65, 0.74) | 0.60 (0.56, 0.64) | 0.86 (0.84, 0.87) |  |
| **Alcohol-drinker** |  |  |  |  |  |  |  |
| No (Never) | 1 (Ref) | 0.85 (0.80, 0.91) | 0.74 (0.70, 0.79) | 0.66 (0.62, 0.70) | 0.51 (0.47, 0.54) | 0.81 (0.79, 0.82) | 0.67 |
| Yes (Past, or current) | 1 (Ref) | 0.87 (0.80, 0.93) | 0.84 (0.77, 0.91) | 0.77 (0.71, 0.84) | 0.65 (0.60, 0.71) | 0.87 (0.85, 0.89) |  |
| **Smoker** |  |  |  |  |  |  |  |
| No (Never) | 1 (Ref) | 0.82 (0.77, 0.88) | 0.77 (0.72, 0.82) | 0.65 (0.61, 0.70) | 0.51 (0.48, 0.55) | 0.80 (0.79, 0.82) | 0.90 |
| Yes (Past, or current) | 1 (Ref) | 0.91 (0.85, 0.97) | 0.80 (0.74, 0.86) | 0.80 (0.74, 0.87) | 0.67 (0.62, 0.74) | 0.88 (0.86, 0.90) |  |
| **Family history of diabetes or CVD** | |  |  |  |  |  |  |
| No | 1 (Ref) | 0.87 (0.83, 0.92) | 0.78 (0.74, 0.82) | 0.70 (0.67, 0.74) | 0.55 (0.52, 0.59) | 0.83 (0.82, 0.84) | 0.43 |
| Yes | 1 (Ref) | 0.64 (0.45, 0.92) | 0.89 (0.64, 1.22) | 0.54 (0.37, 0.79) | 0.52 (0.36, 0.76) | 0.80 (0.72, 0.89) |  |
| **Follow-up time** |  |  |  |  |  |  |  |
| ≤Median | 1 (Ref) | 0.84 (0.78, 0.92) | 0.76 (0.70, 0.83) | 0.70 (0.64, 0.77) | 0.51 (0.46, 0.56) | 0.81 (0.79, 0.83) | 0.70 |
| > Median | 1 (Ref) | 0.88 (0.78, 0.98) | 0.79 (0.70, 0.89) | 0.73 (0.65, 0.83) | 0.57 (0.50, 0.64) | 0.84 (0.81, 0.87) |  |
|  | | | | | | | |
| **Total mortality** | | | | | | | |
| **Age** |  |  |  |  |  |  |  |
| <65 years | 1 (Ref) | 0.85 (0.80, 0.91) | 0.74 (0.69, 0.79) | 0.68 (0.63, 0.73) | 0.68 (0.63, 0.73) | 0.85 (0.83, 0.87) | 0.39 |
| ≥65 years | 1 (Ref) | 0.98 (0.89, 1.07) | 1.01 (0.92, 1.10) | 0.94 (0.86, 1.03) | 0.84 (0.76, 0.92) | 0.94 (0.92, 0.97) |  |
| **Sex** |  |  |  |  |  |  |  |
| Female | 1 (Ref) | 0.80 (0.68, 0.94) | 0.77 (0.66, 0.90) | 0.68 (0.58, 0.80) | 0.59 (0.50, 0.69) | 0.84 (0.80, 0.88) | 0.07 |
| Male | 1 (Ref) | 0.91 (0.86, 0.97) | 0.85 (0.80, 0.90) | 0.81 (0.76, 0.86) | 0.76 (0.72, 0.81) | 0.91 (0.89, 0.92) |  |
| **BMI** |  |  |  |  |  |  |  |
| <25kg/m^2^ | 1 (Ref) | 0.88 (0.80, 0.97) | 0.78 (0.71, 0.86) | 0.74 (0.67, 0.81) | 0.66 (0.61, 0.72) | 0.87 (0.85, 0.89) | 0.67 |
| ≥25kg/m^2^ | 1 (Ref) | 0.89 (0.83, 0.95) | 0.84 (0.79, 0.90) | 0.77 (0.72, 0.83) | 0.71 (0.66, 0.77) | 0.89 (0.87, 0.91) |  |
| **Alcohol-drinker** |  |  |  |  |  |  |  |
| No (Never) | 1 (Ref) | 0.87 (0.81, 0.93) | 0.85 (0.80, 0.91) | 0.78 (0.73, 0.84) | 0.72 (0.67, 0.78) | 0.89 (0.88, 0.91) | 0.20 |
| Yes (Past, or current) | 1 (Ref) | 0.94 (0.87, 1.02) | 0.80 (0.73, 0.88) | 0.82 (0.75, 0.89) | 0.75 (0.68, 0.82) | 0.90 (0.88, 0.93) |  |
| **Smoker** |  |  |  |  |  |  |  |
| No (Never) | 1 (Ref) | 0.82 (0.76, 0.88) | 0.82 (0.76, 0.89) | 0.75 (0.70, 0.81) | 0.70 (0.65, 0.75) | 0.89 (0.87, 0.91) | 0.49 |
| Yes (Past, or current) | 1 (Ref) | 0.98 (0.91, 1.06) | 0.82 (0.75, 0.89) | 0.81 (0.74, 0.89) | 0.75 (0.68, 0.83) | 0.90 (0.88, 0.93) |  |
| **Family history of diabetes or CVD** | | |  |  |  |  |  |
| No | 1 (Ref) | 0.91 (0.86, 0.95) | 0.85 (0.81, 0.89) | 0.82 (0.78, 0.86) | 0.75 (0.71, 0.79) | 0.91 (0.90, 0.92) | 0.47 |
| Yes | 1 (Ref) | 0.67 (0.45, 0.99) | 0.51 (0.34, 0.78) | 0.37 (0.22, 0.62) | 0.46 (0.29, 0.72) | 0.74 (0.66, 0.84) |  |
| **Follow-up time** |  |  |  |  |  |  |  |
| ≤Median | 1 (Ref) | 0.91 (0.86, 0.97) | 0.88 (0.82, 0.94) | 0.83 (0.78, 0.89) | 0.74 (0.69, 0.79) | 0.90 (0.88, 0.92) | 0.88 |
| >Median | 1 (Ref) | 0.85 (0.73, 0.98) | 0.73 (0.62, 0.84) | 0.77 (0.67, 0.90) | 0.80 (0.69, 0.93) | 0.94 (0.91, 0.99) |  |

Data were HRs and 95%CIs among individuals with T2D. Multivariable time-varying cox models were adjusted for age (years), sex (male, female), education (illiteracy or elementary, middle school, college/university), income (< median, ≥ median), marital status (yes, no), alcohol-drinker (never, past, current), family history of diabetes (yes, no), and family history of CVD (yes, no). P_interaction_ was calculated using the Wald test by including interactions between LE8 score and each of the following variables: age, sex, BMI, smoker, alcohol drink, family history of diabetes or CVD, and follow-up time. Given the potential for multiple testing, the statistical level for significance was set at 0.007 (0.05/7 comparisons). LE8, Life’s Essential 8; CVD, cardiovascular disease.

**Supplemental Table 5. Hazard ratios of CVD and mortality per modified time-varying LE8 score by excluding blood glucose**

| **Modified LE8 score** | **Q1** | **Q2** | **Q3** | **Q4** | **Q5** |
| --- | --- | --- | --- | --- | --- |
| **CVD** | 1 (Ref) | 0.88 (0.84, 0.92) | 0.80 (0.76, 0.84) | 0.75 (0.71, 0.79) | 0.61 (0.57, 0.64) |
| **Heart disease** | 1 (Ref) | 0.89 (0.83, 0.95) | 0.84 (0.79, 0.90) | 0.76 (0.71, 0.81) | 0.62 (0.58, 0.67) |
| **Stroke** | 1 (Ref) | 0.85 (0.80, 0.90) | 0.74 (0.69, 0.79) | 0.73 (0.68, 0.78) | 0.56 (0.52, 0.60) |
| **Total morality** | 1 (Ref) | 0.92 (0.87, 0.97) | 0.84 (0.80, 0.89) | 0.87 (0.83, 0.92) | 0.82 (0.77, 0.86) |

Data were HRs and 95%CIs among individuals with T2D. Multivariable time-varying cox models were adjusted for age (years), sex (male, female), education (illiteracy or elementary, middle school, college/university), income (< median, ≥ median), marital status (yes, no), alcohol-drinker (never, past, current), family history of diabetes (yes, no), and family history of CVD (yes, no). CVD, cardiovascular disease; LE8, Life’s Essential 8; T2D, type 2 diabetes

**Supplemental Table 6. Associations of time-varying LE8 with CVD and mortality with additional adjustment for comorbidities and medication**

| **Life’s Essential 8 scores** | **Q1** | **Q2** | **Q3** | **Q4** | **Q5** |
| --- | --- | --- | --- | --- | --- |
| **CVD** | 1 (Ref) | 0.87 (0.83, 0.91) | 0.79 (0.75, 0.83) | 0.72 (0.68, 0.75) | 0.58 (0.55, 0.62) |
| **Heart disease** | 1 (Ref) | 0.87 (0.81, 0.93) | 0.81 (0.76, 0.87) | 0.76 (0.71, 0.82) | 0.61 (0.56, 0.66) |
| **Stroke** | 1 (Ref) | 0.85 (0.80, 0.91) | 0.76 (0.71, 0.81) | 0.66 (0.62, 0.71) | 0.54 (0.50, 0.59) |
| **Total morality** | 1 (Ref) | 0.88 (0.84, 0.93) | 0.82 (0.77, 0.86) | 0.76 (0.72, 0.80) | 0.70 (0.66, 0.74) |

Data were HRs and 95%CIs among individuals with T2D. Multivariable time-varying cox models were adjusted for age (years), sex (male, female), education (illiteracy or elementary, middle school, college/university), income (< median, ≥ median), marital status (yes, no), alcohol-drinker (never, past, current), family history of diabetes (yes, no), family history of CVD (yes, no), hypertension (yes, no), hyperlipidemia (yes, no), hyperglycemia (yes, no), lipid-lowering medications (yes, no), glucose-lowering medications (yes, no), and antihypertensive medications (yes, no). CVD, cardiovascular disease; LE8, Life’s Essential 8; T2D, type 2 diabetes.

**Supplemental Table 7. Associations of time-varying LE8 with CVD and mortality among incident T2D cases diagnosed during follow-up**

| **Life’s Essential 8 scores** | **Q1** | **Q2** | **Q3** | **Q4** | **Q5** |
| --- | --- | --- | --- | --- | --- |
| **CVD** | 1 (Ref) | 0.90 (0.82, 0.98) | 0.76 (0.69, 0.83) | 0.67 (0.61, 0.74) | 0.52 (0.47, 0.57) |
| **Heart disease** | 1 (Ref) | 0.85 (0.75, 0.95) | 0.68 (0.60, 0.77) | 0.67 (0.59, 0.76) | 0.49 (0.43, 0.56) |
| **Stroke** | 1 (Ref) | 0.93 (0.83, 1.04) | 0.80 (0.71, 0.90) | 0.66 (0.59, 0.75) | 0.51 (0.44, 0.58) |
| **Total morality** | 1 (Ref) | 0.87 (0.79, 0.96) | 0.86 (0.78, 0.94) | 0.77 (0.69, 0.85) | 0.62 (0.55, 0.69) |

Data were HRs and 95%CIs among incident T2D cases diagnosed during follow-up only (n=7,732). Multivariable time-varying cox models were adjusted for age (years), sex (male, female), education (illiteracy or elementary, middle school, college/university), income (< median, ≥ median), marital status (yes, no), alcohol-drinker (never, past, current), family history of diabetes (yes, no), family history of CVD (yes, no), and diabetes duration (years). CVD, cardiovascular disease; LE8, Life’s Essential 8 score; T2D, type 2 diabetes.

**Supplemental Table 8. Associations of time-varying LE8 with CVD and mortality among individuals with T2D after excluding cancers**

| **Life’s Essential 8 scores** | **Q1** | **Q2** | **Q3** | **Q4** | **Q5** |
| --- | --- | --- | --- | --- | --- |
| **CVD** | 1 (Ref) | 0.87 (0.83, 0.91) | 0.79 (0.76, 0.83) | 0.71 (0.67, 0.75) | 0.56 (0.53, 0.59) |
| **Heart disease** | 1 (Ref) | 0.86 (0.80, 0.92) | 0.80 (0.75, 0.86) | 0.75 (0.69, 0.80) | 0.58 (0.54, 0.62) |
| **Stroke** | 1 (Ref) | 0.86 (0.80, 0.91) | 0.77 (0.72, 0.82) | 0.66 (0.61, 0.70) | 0.53 (0.49, 0.57) |
| **Total morality** | 1 (Ref) | 0.87 (0.82, 0.93) | 0.80 (0.75, 0.85) | 0.75 (0.70, 0.79) | 0.66 (0.62, 0.70) |

Data were HRs and 95%CIs among individuals with T2D after excluding cancers cases (n=18,827). Multivariable time-varying cox models were adjusted for age (years), sex (male, female), education (illiteracy or elementary, middle school, college/university), income (< median, ≥ median), marital status (yes, no), alcohol-drinker (never, past, current), family history of diabetes (yes, no), and family history of CVD (yes, no). CVD, cardiovascular disease; LE8, Life’s Essential 8; T2D, type 2 diabetes.

**Supplemental Table 9. Associations of time-varying LE8 after excluding CVD and deaths within the first 4-year follow-up**

| **Life’s Essential 8 scores** | **Q1** | **Q2** | **Q3** | **Q4** | **Q5** |
| --- | --- | --- | --- | --- | --- |
| **CVD** | 1 (Ref) | 0.88 (0.83, 0.93) | 0.78 (0.74, 0.82) | 0.70 (0.66, 0.73) | 0.54 (0.51, 0.57) |
| **Heart disease** | 1 (Ref) | 0.90 (0.84, 0.97) | 0.81 (0.75, 0.87) | 0.74 (0.68, 0.80) | 0.57 (0.53, 0.62) |
| **Stroke** | 1 (Ref) | 0.85 (0.80, 0.92) | 0.74 (0.69, 0.80) | 0.64 (0.60, 0.69) | 0.50 (0.46, 0.54) |
| **Total morality** | 1 (Ref) | 0.92 (0.87, 0.98) | 0.82 (0.77, 0.87) | 0.79 (0.75, 0.84) | 0.73 (0.69, 0.78) |

Data were HRs and 95%CIs among individuals with T2D after excluding incident CVD and deaths within the first 4-year of follow-up (n=18,452). Multivariable time-varying cox models were adjusted for age (years), sex (male, female), education (illiteracy or elementary, middle school, college/university), income (< median, ≥ median), marital status (yes, no), alcohol-drinker (never, past, current), family history of diabetes (yes, no), and family history of CVD (yes, no). CVD, cardiovascular disease; LE8, Life’s Essential 8; T2D, type 2 diabetes.

**Supplemental Table 10. Associations of time-varying LE8 and LS7 with the outcomes among individuals with T2D**

| **Life’s Essential 8 score** | **Low CVH**  **(0-49 points)** | **Moderate CVH**  **(50-79 points)** | **High CVH**  **(80-100 points)** |
| --- | --- | --- | --- |
| **CVD** | 1 (Ref) | 0.74 (0.71, 0.77) | 0.42 (0.36, 0.49) |
| **Heart disease** | 1 (Ref) | 0.76 (0.73, 0.80) | 0.52 (0.43, 0.63) |
| **Stroke** | 1 (Ref) | 0.70 (0.67, 0.74) | 0.32 (0.25, 0.40) |
| **Total morality** | 1 (Ref) | 0.82 (0.79, 0.85) | 0.66 (0.57, 0.75) |
| **Life’s Simple 7 score** | **Poor CVH** | **Intermediate CVH** | **Ideal CVH** |
|  | **(0-7 points)** | **(8-11 points)** | **(12-14 points)** |
| **CVD** | 1 (Ref) | 1.00 (0.96, 1.04) | 0.65 (0.56, 0.75) |
| **Heart disease** | 1 (Ref) | 1.05 (0.99, 1.10) | 0.67 (0.55, 0.82) |
| **Stroke** | 1 (Ref) | 0.96 (0.91, 1.01) | 0.62 (0.50, 0.75) |
| **Total morality** | 1 (Ref) | 1.02 (0.99, 1.06) | 0.67 (0.58, 0.77) |

Data were HRs and 95%CIs among individuals with T2D. Multivariable time-varying cox models were adjusted for age (years), sex (male, female), education (illiteracy or elementary, middle school, college/university), income (< median, ≥ median), marital status (yes, no), alcohol-drinker (never, past, current), family history of diabetes (yes, no), family history of CVD (yes, no), and sleep (only for Life’s Simple 7). According to the AHA’s recommendation, Life’s essential 8 score was divided into low, moderate, and high CVH groups and Life’s Simple 7 score was divided into poor, intermediate, and ideal CVH groups. CVD, cardiovascular disease; CVH, cardiovascular health; LE8, Life’s Essential 8; T2D, type 2 diabetes.

**Supplemental Figure 1. Selection of participants with diabetes in the analysis**

LE8, life’s essential 8; CVD, cardiovascular disease.


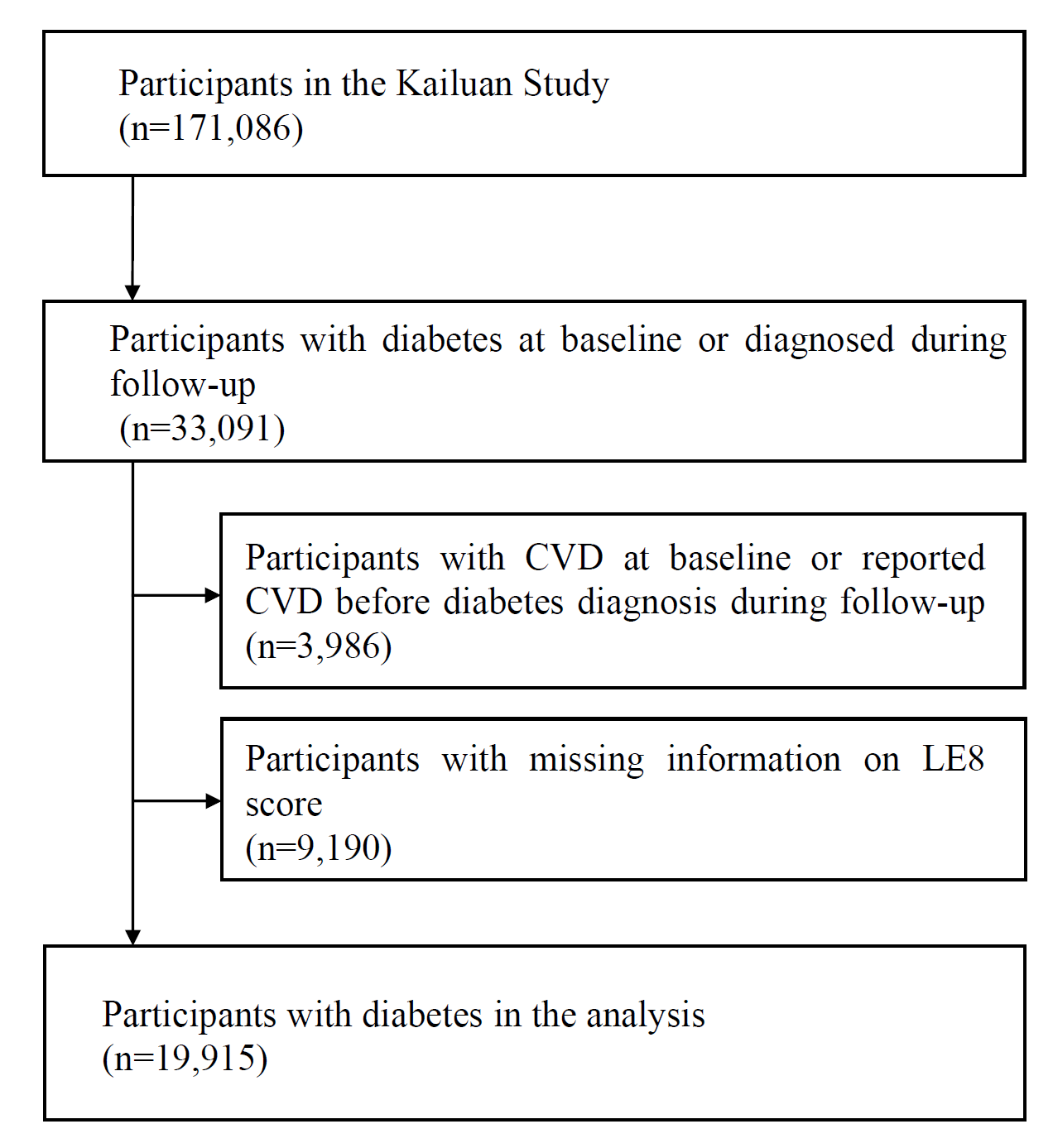

Supplement: Supplemental Tables 1–10 and Supplemental Figure 1 [file mmc1.docx]
